# Extracellular Vesicles in Multiple Sclerosis: What are They Telling Us?

**DOI:** 10.3389/fncel.2014.00100

**Published:** 2014-03-28

**Authors:** Matías Sáenz-Cuesta, Iñaki Osorio-Querejeta, David Otaegui

**Affiliations:** ^1^Multiple Sclerosis Unit, Neuroscience Area, Biodonostia Health Research Institute, San Sebastián, Spain; ^2^Spanish Network on Multiple Sclerosis, Madrid, Spain

**Keywords:** extracellular vesicle, exosomes, microvesicle, multiple sclerosis, biomarker, therapy

## Abstract

Extracellular vesicles (EVs) are membrane-bound particles secreted by almost all cell types. They are classified depending on their biogenesis and size into exosomes and microvesicles or according to their cell origin. EVs play a role in cell-to-cell communication, including contact-free cell synapsis, carrying active membrane proteins, lipids, and genetic material both inside the particle and on their surface. They have been related to several physiological and pathological conditions. In particular, increasing concentrations of EVs have been found in many autoimmune diseases including multiple sclerosis (MS). MS is a central nervous system (CNS) demyelinating disease characterized by relapsing of symptoms followed by periods of remission. Close interaction between endothelial cells, leukocytes, monocytes, and cells from CNS is crucial for the development of MS. This review summarizes the pathological role of EVs in MS and the relationship of EVs with clinical characteristics, therapy, and biomarkers of the disease.

## What are Extracellular Vesicles?

Extracellular vesicles (EVs) are membrane-bound particles coming from inside a cell or formed directly from its membrane, and excreted to the extracellular medium, that carry information whose function is cell-to-cell communication without direct contact. They play a role in physiological and pathological conditions, being released during cell activation, stress, and apoptosis. Specifically, these vesicles carry proteins, lipids, and genetic materials such as DNA, RNA, and miRNA, producing genotypic (Waldenström et al., 2012) and phenotypic (van der Vos et al., 2011) modifications in the recipient cell. This is facilitated by the receptors on the surface of the EV membrane that allow the target cell to identify the vesicles and interact with them (Choudhuri et al., 2014).

## EVs Classification

### Biogenesis

Though there are several ways of classifying EVs, the main division in nomenclature is based on biogenesis. Those formed inside multivesicular bodies and released extracellularly upon fusion of these bodies with the plasma membrane are called exosomes (Théry et al., 2009). Their main characteristic is to have a uniform size of between 30 and 150 nm, making them the smallest EVs. On the other hand, those known as microparticles (MP), microvesicles (MV), or ectosomes come from the modification of the cell membrane after external or internal stimuli, leading to a softening of the membrane-adjacent structure and allowing evagination and vesicle formation followed by fission on the connecting membrane stalks until their full detachment. These MV/MPs vary greatly in size, ranging from 0.3 to 1 μm in diameter (Mause and Weber, 2010; Frey and Gaipl, 2011; Lai and Breakefield, 2012). However, the current trend is to call the entire set EVs, the term used by the newly formed International Society of Extracellular Vesicles (Witwer et al., 2013).

In this review, we follow this trend, using the term EVs to refer to all vesicles; we note, however, that specifically in multiple sclerosis (MS) related-research most studies refer to them as MPs or MVs.

### Cell origin

Extracellular vesicles have been also classified as a function of their cell origin depending on the parental cell from which they arose, so far the most studied being those obtained from circulating cells in peripheral blood. Each cell has characteristic markers on its membrane enabling subsequent identification of the EV, e.g., as erythrocyte-, leukocyte-, platelet-, endothelial-, or monocyte-derived. Further, studies focusing on central nervous system (CNS)-derived EVs have described neural stem cell-, neuron-, astrocyte-, microglia-, and oligodendrocyte-derived vesicles (Lai and Breakefield, 2012) with the goal of finding markers that may reflect CNS status, since they can be detected remote from the site of release after cell activation.

## Techniques for Studying EVs

The study of EVs is not straightforward, particularly with respect to isolation and characterization due to their small size and the low concentrations found in human fluids. Further, although efforts have been made to unify criteria in EV research (Robert et al., 2008; Dey-Hazra et al., 2010; Lacroix et al., 2012a; Witwer et al., 2013), they are not yet clearly established, making it difficult to compare studies. Differences derived from centrifugation protocols, fluorochrome labeling, and gating strategies represent as yet unsolved barriers to standardization. Nevertheless, the most widely used techniques can be summarized as follows.

### Isolation

The main approach that has been used for isolating EVs from human fluids or culture media supernatants is a series of sequential centrifugation steps. Different purities are obtained depending on the number of steps completed. Briefly, a first centrifugation step at a low velocity (200–300 g) separates cells from EV-containing fluid, which can be further purified or directly pelleted. For a further purification, a second centrifugation must be carried out (at 2,000–10,000 × *g*, depending on the fluid or EV fraction required). Otherwise, EVs can be directly pelleted from the first supernatant (centrifuging at forces of 10,000 up to 100,000 × *g*). Though there are many variations among authors, the first approach to EV analysis is usually based on the aforementioned steps. As an alternative protocol to obtain a more pure EV fraction, a sucrose gradient can be combined with one of the centrifugation steps.

Another isolation technique is polymeric precipitation (e.g., Exoquick, System Biosciences, CA, USA). The main advantage of this approach is rapid sample processing. However, the low purity obtained and mixing of different EV subsets make results difficult to interpret.

The extraction of EVs by passing a sample through filters is a cheap and easy method that can be applied alone or combined with centrifugation. There is, however, a risk of contamination with particles other than EVs of the same size.

### Characterization

Flow cytometry is the technique most widely employed for studying EVs (including in MS research) to the possibility of using multiple parameters to identify the same vesicle. It is a powerful characterization tool, the process is rapid and the results can be quantified. Its main limitation is poor discrimination under 0.5 μm. However, new high-resolution cytometers can detect particles as small as 0.2–0.3 μm.

Recently, two novel tools appeared on the market created to characterize nanoparticles in size and concentration with a high resolution. They measure particles based on tunable resistive pulse sensing (qNANO, IZON Science, New Zealand) and Brownian motion of the particle with nanoparticle tracking analysis software (NS500 and NS300, Nanosight, UK). The simple and user-friendly operation and powerful measurements provided by these instruments herald a new era in the analysis of EVs.

**Figure 1:**
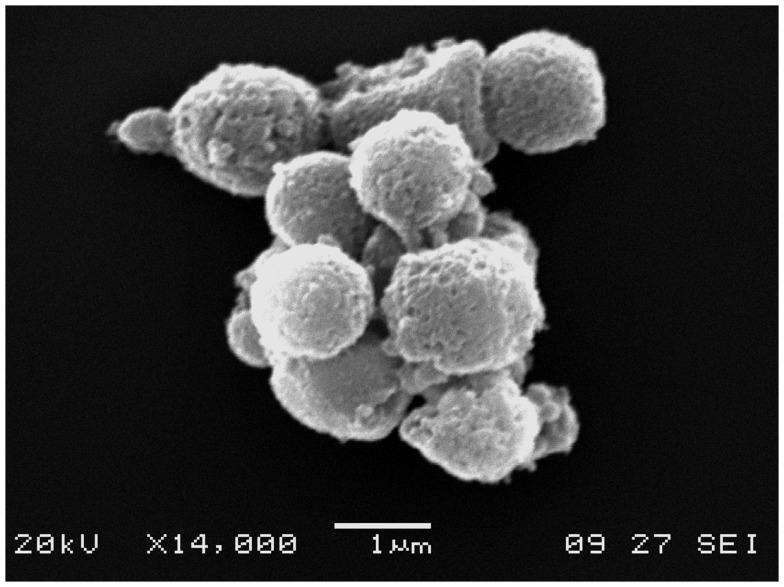
**Electron microscopy image of EVs**. An electron microscopy image of an EV cluster obtained from peripheral blood. Note the rounded shape and cell membrane-like appearance of EV surfaces.

Electron microscopy is usually performed in combination with flow cytometry to provide direct evidence of the presence of EVs, and it provides what is arguably the highest quality morphological information (Figure 1). On the other hand, the expensive and complex processing of samples limits its use.

Fluorescence microscopy is normally used to analyze EV function *in vitro*, as well as to localize EV in tissues and budding process. In particular, confocal microscopy is widely used in EV research.

In addition, enzyme-linked immunosorbent and Western blot assays have also been employed for analysis of EVs but are less extensively used due to the poor characterization they provide and that they are difficult to quantify, respectively.

Notably, next-generation sequencing techniques are currently expanding to the field of EVs, specifically in the attempt to characterize their genetic cargo.

## EVs in Neuroscience

Vesicle secretion and the transfer of material carried within them in the CNS under physiological conditions were described many decades ago (de Robertis and Bennett, 1954). The classic example was the presence of vesicles in the neuronal synapses (de Robertis and Bennett, 1955). However, the mechanisms involved and modulation thereof by astrocytes, through the release of vesicles into the synaptic space, have only been properly understood in recent years (Antonucci et al., 2012). Vesicles have been implicated not only in the propagation of signals, but also in controlling neurogenesis with exosomes being involved in the regulation of myelin membrane biogenesis (Marzesco et al., 2005; Bakhti et al., 2011) and repairing damaged neurons (Court et al., 2011). Moreover, a recent study identified a new mechanism of regulation of the axonal integrity mediated by oligodendrocyte-derived EVs transferred to neurons (Frühbeis et al., 2013). It has been observed that EVs are released by neural cells, oligodendrocytes, neurons, microglia, astrocytes in the brain, and Schwann cells in the peripheral nervous system (reviewed by Lai and Breakefield, 2012; Frühbeis et al., 2012). All this implies that EVs perform functions necessary for growth and normal functioning of the nervous system.

In addition, EVs are involved in processes of CNS diseases carrying specific pathological cargo or performing functions that produce potential damage (Lai and Breakefield, 2012). Several studies have found variations in the number and function of circulating EVs in peripheral blood in diseases including Alzheimer’s disease, dementia, epilepsy, stroke, traumatic brain injury, malaria, and tumors (mainly glioblastoma), among others (reviewed by Lai and Breakefield, 2012; Doeuvre et al., 2009). To explore these functions, most studies expose primary cell cultures to suspensions of EVs analyzing the effects produced by EV in the cells such as morphological changes, fusion processes, induction of proliferation, and apoptosis. Another approach is to analyze EVs derived directly from human fluids. For this, peripheral blood and CSF are the most frequently studied samples. On the other hand, few studies have explored whether variations in EVs in CSF directly reflect the pathophysiology of the CNS (Morel et al., 2008; Huang et al., 2009; Street et al., 2012; Verderio et al., 2012; Mobarrez et al., 2013; Patz et al., 2013; Joshi et al., 2014) and only a couple of them have examined EVs derived from brain cells obtained from the CSF as a surrogate marker for what occurs in the CNS (Verderio et al., 2012; Joshi et al., 2014). Above all, it has not yet been elucidated whether EVs are able to migrate from the blood across the blood–brain barrier (BBB) into the CNS (or not) and vice versa (Smalheiser, 2009). More studies are required to provide evidence on whether there is an EV-mediated communication channel between the nervous and the cardiovascular systems.

## Multiple Sclerosis (MS) as a Neuroimmune Disease

Multiple sclerosis is a chronic autoimmune disease affecting the CNS, the cause of which remains elusive. It is, however, established that the pathogenesis of the disease involves genetic, environmental, and immune components (Bernard and Kerlero de Rosbo, 1992). There are different clinical forms, but the most prevalent is relapsing–remitting MS, characterized by outbreaks of symptoms lasting 1–3 weeks called relapses, followed by a recovery phase. During relapses, multiple areas of demyelination emerge, this being the main pathological feature of the disease. Immune activation involved in the onset of the disease causes a release of proinflammatory cytokines (TNF, IL1-beta, IFN-gamma) plus a proliferation of leukocytes, monocytes, and platelets (Martino and Hartung, 1999). At the same time, endothelial dysfunction of the BBB affects its permeability, facilitating the activation, adhesion, and transendothelial migration of monocytes and T-lymphocytes into the CNS (Minagar et al., 2012). Cytokines and chemokines released at the site of a lesion recruit glial cells, macrophages, and lymphocytes perpetuating the immune activation leading to a chronic inflammatory state (McFarland and Martin, 2007). Currently, the diagnosis of MS is based on the 2010 revised McDonald criteria (Polman et al., 2011) including careful clinical evaluation supported by MRI findings and oligoclonal banding in the CSF, the main complementary tools. The treatment of MS has undergone a revolution with the advent of IFN-beta as a treatment in the 1980s and more recently with the new immunomodulator drugs, such as natalizumab and fingolimod.

Several studies summarized in this review suggest EVs are active players in the pathophysiological development of this disease. More specifically, higher numbers of EVs have been observed in MS patients than in healthy controls and a role for EVs has been proposed in inflammatory progression and lesion repair. Because of this, they could serve as new biomarkers of disease development and targets for future treatments.

We will discuss these issues in the following sections. In Table 1 we summarize the origins and makers used for EVs reported.

**Table 1:** **Cellular origins of extracellular vesicles (EVs) in multiple sclerosis research**.

EV origin	Marker	Sample	Technique	Reference
Endothelial	CD31+/CD42−	PPP and MVEC	FC	Minagar et al. (2001)
		WB and MVEC	FC	Jy et al. (2004), Jimenez et al. (2005)
	CD51	PPP and MVEC	FC	Minagar et al. (2001)
	CD54	WB and MVEC	FC	Jy et al. (2004), Jimenez et al. (2005)
	CD106	WB and MVEC	FC	Jy et al. (2004)
	CD62E	WB and MVEC	FC	Jy et al. (2004), Jimenez et al. (2005)
	CD146	PPP	FC	Lowery-Nordberg et al. (2011)
Platelet	CD61	PFP	FC	Sáenz-Cuesta et al. (2014)
	CD41	PPP	FC	Sheremata et al. (2008)
Leukocyte	CD45	PFP	FC	Sáenz-Cuesta et al. (2014)
Monocyte	CD14	PFP	FC	Sáenz-Cuesta et al. (2014)
Astrocyte	GFAP	CSF	FM/WestB	Verderio et al. (2012)
Neuronal	SNAP-25	CSF	FM/WestB	Verderio et al. (2012)
Oligodendrocyte	MBP	CSF	FM/WestB	Verderio et al. (2012)
Microglia/macrophage	IB4	CSF	FM/FC/EM	Verderio et al. (2012)
**GENERAL MARKERS**				
Exosomes	CD63	PFP	WestB	Williams et al. (2013), Gatson et al. (2011)
Microvesicles	AnV	CSF	FC	Verderio et al. (2012)

*EM, electron microscopy (immunogold); PPP, platelet poor plasma; PFP, platelet free plasma; WB, whole blood; WestB, Western blot; CSF, cerebrospinal fluid; FC, flow cytometry; FM, fluorescence microscopy; MVEC, microvascular endothelial cell culture*.

## Immune Roles of EVs in MS

One of the necessary processes for the establishment of MS is the transendothelial migration of leukocytes into the CNS through the BBB. This migration is favored by a weakening of the barrier. The fact that this mechanism is crucial to the pathogenesis of MS is demonstrated by the benefits observed with natalizumab, which blocks the entry of leukocytes into the CNS (del Pilar Martin et al., 2008). Proinflammatory cytokines such as TNF-alpha, IFN-gamma, and IL1-beta released by inflammatory cells mediate the breaching of the BBB by the upregulation of the expression of adhesion molecules (VCAM-1, E-selectin, and PECAM-1) (Dore-Duffy et al., 1995), the loss of junctional integrity (Minagar et al., 2003), and the release of endothelial-derived EVs (EEVs) (Minagar et al., 2001). EEVs from the endothelial cells of BBB and other EVs shed from surrounding cells [leukocytes (LEV), platelets (PEV), microglia (MEV), and astrocytes] are vectors of numerous agents carried inside these vesicles or bound to their plasma membrane. The presence of metalloproteinases in EV cargo suggests that they may participate in the degradation of the extracellular matrix involved in BBB disruption (Sbai et al., 2010; Lacroix et al., 2012b). Moreover, caspase 1 carried by EVs shed by monocytes and microglia has been shown to regulate proteolytic activity of metalloproteases on endothelial cells (Bianco et al., 2005; Sarkar et al., 2009).

**Figure 2:**
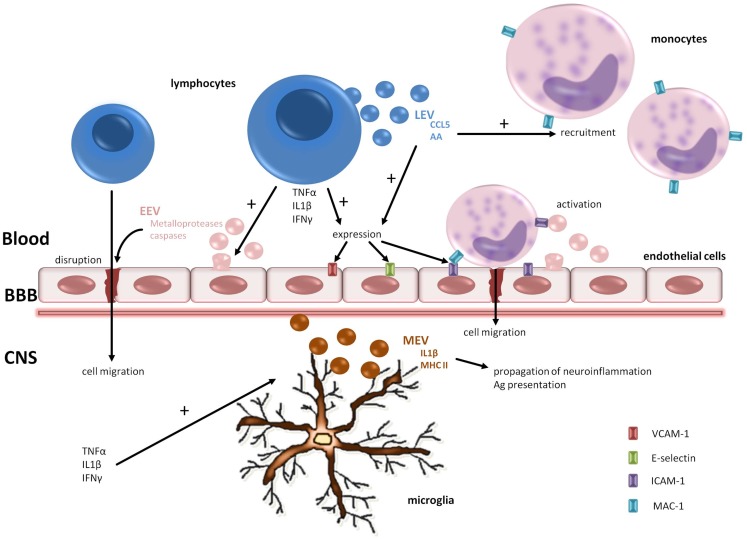
**Pathogenic roles of EV in MS**. EVs are involved in the transendothelial cell migration of lymphocytes and monocytes and the spread of neuroinflammation. Metalloproteases carried by EEVs promote BBB disruption. The release of proinflammatory cytokines from lymphocytes augments adhesion molecules on endothelial cells facilitating cell adhesion. In the CNS compartment, microglia play a key role in propagation of neuroinflammation shedding MEVs containing IL1-b and MHC-II. BBB, blood–brain barrier; CNS, central nervous system; AA, arachidonic acid; EEV, endothelial-derived extracellular vesicle; LEV, leukocyte-derived extracellular vesicle; MEV, microglia-derived extracellular vesicle.

Minagar et al. (2001) hypothesized that plasma from MS patients contains factors that can induce endothelial activation, as suggested by the release into circulation of CD31+ EEVs from microvascular endothelial cell culture (MVEC) – a BBB model – treated with plasma from patients both in exacerbation and remission. After this pivotal study, Jy et al. (2004) demonstrated that EEVs found in plasma are able to interact and form complex with monocytes and induce their activation. These activated monocytes express Mac-1 integrin, which is an ICAM-1 receptor. The union of Mac-1 of monocytes with ICAM-1 of endothelial cells plays an important role in the transendothelial migration of inflammatory cells. Moreover, activated T cells release EVs containing the chemokine CCL5 and arachidonic acid responsible for promoting recruitment of monocytes and upregulating ICAM-1 in endothelial cells and LFA1 and Mac-1 in monocytes (Barry et al., 1998). To sum up, these data suggest that EEVs shed from the activated endothelial cells in MS patients promote the migration of monocytes and lymphocytes through the BBB and assist with the formation of demyelinating lesions. A validation of this hypothesis was performed in an elegant experiment carried by Jimenez et al. (2005): they investigated the transendothelial migration of monocytes using the MVEC model, adding plasma from remitting or relapsing MS patients and controls, and found that only the plasma from patients in relapse significantly promoted transendothelial migration. See Figure 2 for a graphical summary of this paragraph.

Shedding new light on the role of EVs, a few recent studies have investigated EVs in the animal model of MS called experimental autoimmune encephalitis (EAE). The first published by Gatson et al. (2011) analyzed EVs (exosomes in this case) in late pregnant compared to virgin EAE mice. Results showed that EVs derived from serum of mice in late pregnancy were more numerous than those isolated from virgin mice. The proliferation of T-cells derived from splenocytes was also explored in the presence of whole serum, purified EVs, and EV-depleted serum. The three phases derived from pregnant animals were significantly more suppressive of T-cell proliferation than EVs from virgin animals or cells cultured without any EVs. On the basis of these findings, authors concluded that EVs are responsible for immune modulation during EAE pregnancy. A further study by the same group analyzed this immune modulation showing a reduction in IFN-gamma production and expression of Tbet (Th1 transcription factor) in T cells exposed to pregnancy-derived EVs. In addition, these researchers demonstrated the effect of pregnancy-derived EVs on migration to lesion areas in EAE of oligodendrocyte precursor cells and their maturation (Williams et al., 2013). This is the first publication that denoted a protective role of EVs in MS/EAE.

Verderio et al. (2012) identified other EV origins analyzing in depth CSF from humans and mice, both healthy and MS/EAE. Several types of brain cell including neurons, astrocytes, and resident microglial cells give rise to EVs. Peripheral macrophages are virtually absent in healthy brain parenchyma suggesting that myeloid EVs obtained in CSF are derived from resident microglia in the normal brain. This group also revealed that microglia store and release IL1-beta and MHC-II suggesting that the EVs produced from reactive myeloid cells may propagate neuroinflammation and provide an efficient route for rapid dissemination and presentation of antigens.

Regarding platelet-derived EVs (PEVs) in MS, P-selectin was observed on PEVs capable of binding to PSGL-1 and PECAM-1 from lymphocytes by increasing the expression of integrins such as α4β1 (VLA-4), promoting the binding of these cells to the endothelium (Sheremata et al., 2008). Interestingly, this epitope is the target of natalizumab, one of the recent therapies approved for relapsing–remitting MS.

All this evidence supports the idea that EVs are involved in MS playing a pathological role, acting as immunomodulator agents in the disruption of the BBB and the propagation of inflammation of the parenchyma but that, on the other hand, they contribute to the repair of demyelinating lesions.

## Are EVs Reliable Biomarkers in MS?

As stated above, the association between EV concentration and the pathological condition of MS patients is clearly established. The next challenge is to develop the application of EVs as useful biomarkers: as well as providing relevant information, they are easy to process at a low cost and hence their use could be extended to large study populations. However, clearly, the adoption of EVs as biomarkers needs to be based on an objective assessment of their diagnostic and monitoring potential for the disease in question. In the case of MS, EV measurements must be correlated with the clinical judgment of the neurologist, established scores, and the results of other complementary tests such as MRI. Several studies discussed in the following paragraphs have addressed these issues but it should be noted that the results are mixed, sometimes inconsistent, depending on the type of EV (MV, MP, or exosomes), their cell origin, methods employed, and analysis performed.

### EV concentration and clinical status

A relationship between EV counts in plasma and MS status was first proposed by Minagar et al. (2001) more than a decade ago. Their results revealed that CD51+ EEV concentrations were higher in relapse and remission, while those of CD31+ EEVs were only higher during relapse, compared to healthy controls. They proposed that the increase in CD51+ EEVs was related to chronic inflammation owing to endothelial erosion with subendothelial matrix exposure, and that CD31+ EEVs reflect acute endothelial damage. This was tested *in vitro*, and the results were only partially reproduced. Together the findings pointed to the existence of factors present in the plasma of MS patients but not in the *in vitro* model, such as activated leukocytes present during exacerbations, which were able to regulate the release of EEVs. Authors also described a concordance between CD31+ EEV counts and gad+ MRI findings. They claimed that these vesicles were as sensitive as gad+ MRI for detecting disease activity, and also that a decrease in vesicle count could precede a negativization of MRI findings. However, this was criticized for being a premature speculation and not supported by sufficient evidence (Larkin, 2001).

In the same line of research, 3 years later Jy et al. (2004) explored whether CD54+ and CD62E+ EEVs bound leukocytes *in vitro* and in whole blood from MS patients and controls. Their main conclusion was that CD54+ EEVs form complexes with monocytes in a TNF-alpha environment and also activated them. CD62E+ EEV–monocyte complexes were more numerous during exacerbations than in remission while the number of CD54+ EEV–monocyte complexes remained unchanged, suggesting that the former would be a better marker for monitoring MS. Authors reported that the measurement of both EEV–monocyte complexes together as a single EEV–monocyte complex fraction appeared to be more sensitive to MS exacerbation than gad+ MRI and even more sensitive than the CD31+ EEV analysis studied in their previous work. Finally, free EEVs (unbound to cells) bearing CD62E allowed better discrimination of disease activity (relapsing vs. remitting patients) than CD54+ EEVs, but not compared to the previously reported CD31+ EEVs.

Conversely a year later, Jimenez et al. explored free CD54+ and CD62E+ EEVs *in vitro* reporting an increase in both markers during relapse and normal values similar to control in remission. Analyzing EEVs from relapsing patients only, CD54+ and CD62E+ phenotypes were present in significantly higher numbers than CD31+ EEVs, indicating that they were more sensitive (*in vitro*) for identifying MS status (Jimenez et al., 2005). As stated by Witwer et al. (2013), EV studies are highly heterogeneous, this being attributable to the lack of standardized methods. Possibly, this underlies the mixed results described here, particularly with respect to the earlier work by Jy and colleagues.

Besides EEVs, other EVs have been explored including those derived from platelets, leukocytes, and monocytes. Platelet activation in patients with MS may be secondary to endothelial damage (Sheremata et al., 2008). CD62P (P-selectin) levels have been shown to be higher in MS patients than controls. Twofold higher CD41+ PEV counts were found in MS patients compared to controls, these vesicles showing properties as anticoagulants (Sheremata et al., 2008). Our group also demonstrated a significant difference in CD61+ PEV, CD45+ LEV, and CD14+ MEV counts in samples from MS patients compared to those from healthy controls (Sáenz-Cuesta et al., 2014). Moreover, the PEV count was found to be higher in untreated MS patients than controls. Relapsing–remitting patients had the highest counts for the three subtypes of EVs while secondary progressive patients were found to have similar numbers to those in healthy controls. We hypothesized that EVs reflect disease status with more being shed during inflammatory periods and numbers returning to baseline during chronic progressive degeneration. Another approach to monitoring the progression of the disease is to assess patient’s disability using the Expanded Disability Status Scale. Our group found no relationship, however, between EV counts and scores on this scale, and nor were the counts related to disease duration or patients’ age.

In human CSF, the numbers of EVs have also been seen to be higher in patients than controls (Verderio et al., 2012). Among patients, the acute phase was associated with higher numbers of MEVs than stable or chronic phases. In addition, MEVs counts correlated linearly with gad+ MRI images. In line with this, the concentration of MEVs obtained from CSF of EAE mice reflects the course and severity of EAE. The absolute numbers of MEVs were closely associated with the course of the disease, peaking at onset and during clinical relapses, and decreasing in the chronic phase of the disease or stable phase. In this work, authors also explored the potential of MEVs as a possible biomarker in MS plotting ROC curves. Specifically, based on ROC analysis, they obtained a sensitivity of 85% and specificity of 100% for distinguishing clinically isolated syndrome patients from healthy controls, and a sensitivity of 82% and specificity of 82% for differentiating stable (relapse-free patients) from relapsing MS patients.

### EVs and MS therapy

Current MS therapy is based on the modulation of the immune system with a wide range of drugs. In some cases, including IFN-beta, natalizumab, and fingolimod, the effect of the drug on EVs has been explored. However, there are several new drugs, already approved (teriflunomide, alemtuzumab, BG-12) or in the final phases of testing (laquinimod, alemtuzumab, ocrelizumab), in which the potential modulation of EVs has not yet been investigated.

IFN-beta has antiviral and immunoregulatory activity mediated by its interaction with specific cell receptors on the surface of human cells. The precise mechanism of action in MS is still under investigation. So far, it is known that IFN-beta reduces the permeability of the BBB inhibiting leukocyte migration to the CNS (Calabresi et al., 1997) possibly interfering with endothelial adhesion, shifting the cytokine balance from Th1 to Th2, and increasing the expression of occludin at endothelial tight junctions (Dhib-Jalbut et al., 1996).

The effect of IFN-beta 1b on EVs was first explored by Jimenez et al. (2005) who observed an inhibitory effect on EEV production *in vitro* from MVEC culture adding plasma from MS patients, both in remission and relapse. Moreover, it was shown that monocyte–EEV complex formation and transendothelial migration are impaired after IFN-beta 1b exposure.

A first prospective study in a cohort with relapsing–remitting MS revealed a reduction in the numbers of CD31+ EEVs in plasma from week 12 of treatment with IFN-beta 1a (Sheremata et al., 2006). Conversely, no correlation was found with MRI, though there was insufficient data to draw definitive conclusions. Findings in a second cohort treated with high doses of INF-beta 1a and followed-up for a year suggest that CD54+ EEV number represents a more sensitive marker of treatment effect than CD31+ or CD146+ EEV numbers, while results showed a correlation of both CD31+ and CD54+ EEVs with T1-weighted MRI findings (the relation with CD146+ EEV failing to reach statistical significance) (Lowery-Nordberg et al., 2011). Authors speculate that the decrease they observed in plasma vesicles with IFN-beta therapy reflects a reduced interaction between CD4+ T-cells and the endothelium and subsequently less migration of the cells through a restored BBB.

Another immunomodulating drug approved (in 2006) for MS treatment is natalizumab, a recombinant humanized monoclonal antibody. A selective adhesion molecule inhibitor, binds to the alpha-4 subunit of human integrins profusely expressed on the surface of all leukocytes except neutrophils. In particular, it binds to alpha-4-beta-1 integrin, blocking the interaction with its analog receptor, the vascular cell adhesion molecule-1 (VCAM-1). Disruption of these molecular interactions prevents mononuclear leukocyte migration across the endothelium into the inflamed parenchymal tissue (Selewski et al., 2010). In a recent study analyzing plasma PEVs, LEVs, and MEVs, our group found higher counts of all three EV subtypes in IFN-beta and natalizumab-treated than untreated patients (Sáenz-Cuesta et al., 2014). No significant differences were found between the two therapies. A plausible hypothesis specifically for the rise in LEV number in natalizumab-treated patients is that blockage of leukocyte entry into the CNS would result in increase in the number of leukocytes in the blood compartment and, in turn, of LEVs in particular. The rise observed in the other two EV subtypes is, however, less well-understood.

Fingolimod is a new oral immunomodulator drug approved for the relapsing–remitting form of MS. It binds and induces downregulation of the sphingosine 1 phosphate receptors present in lymphocytes regulating their egress from lymphoid tissues into the circulation. In that way, the drug reduces autoaggressive lymphocyte infiltration into the CNS (Chun and Hartung, 2010). Acid sphingomyelinase (aSMase) is inhibited by fingolimod (Dawson and Qin, 2011) and this enzyme controls EV production. These observations led Verderio et al. to theorize that using fingolimod could inhibit MEV shedding from reactive microglia and also macrophage infiltration into the CNS. Their experiments in an EAE model confirmed that MEV numbers decreased to baseline levels in the CSF with the administration of fingolimod. In mice, symptom scores and MEV counts were correlated during fingolimod treatment. Hence, a novel effect of fingolimod was postulated, namely, that it limits the spreading of the inflammatory signal by impairment of MEV production (Verderio et al., 2012). Despite these conclusions, there have so far been no reports evaluating the effect of fingolimod on EVs in humans, probably because it has only relatively recently become available commercially.

Apart from being biomarker for treatment response, it has been proposed that engineered EVs be loaded and used to deliver exogenous compounds for therapeutic purposes, raising the prospect of a novel clinical application for EVs. Preliminary studies with exosomes have been carried out in some types of cancer (Kosaka et al., 2013); however, more research is required before this approach can be used in clinical practice as a complementary therapy. Particularly in MS, a recently published study explored the ability of exosomes packed with microRNA to increase baseline myelination, reduce oxidative stress, and improve remyelination (Pusic et al., 2014). The results showed a significant increase in myelination in hippocampal slice cultures. Nevertheless, the effects of this therapeutic approach need to be investigated further, first in an animal model such as EAE and later in a clinical trial.

## Concluding Remarks

Extracellular vesicles play important roles in the development of MS, in particular activating cells during relapses, leading to migration through the BBB, and spreading inflammation in CNS tissue. On the other hand, a protective effect of EVs has been described with the induction of maturation and migration of oligodendrocyte precursor cells.

Regarding the application of EV research findings to daily clinical practice, it is not yet possible to propose EVs as a specific biomarker for MS due to no compounds having been sufficiently closely linked to the disease. Nevertheless, there is evidence that they reflect disease progression. Particularly, EEVs, PEVs, and MEVs appear to be the most accurate markers. What is more, the effects of treatments seem to be reflected in EV counts. We consider it likely that new carefully designed studies with longer follow-up periods will allow us to confirm the involvement of EVs suggested by our current knowledge and open future applications.

Finally, there is an urgent need for consensus guided by the new scientific societies for EVs to standardize the methodologies and instruments used, in the analysis of EVs with potential applications in clinical practice, and thereby make it possible to obtain comparable results.

## Conflict of Interest Statement

The authors declare that the research was conducted in the absence of any commercial or financial relationships that could be construed as a potential conflict of interest.

## Abbreviations

BBB, blood–brain barrier; CNS, central nervous system; CSF, cerebrospinal fluid; EAE, experimental autoimmune encephalitis; EEV, endothelial-derived EV; EV, extracellular vesicle; LEV, leukocyte-derived EV; MEV, monocyte/microglia-derived EV; MP, microparticle; MS, multiple sclerosis; MV, microvesicle; PEV, platelet-derived EV.
